# An Integrated Spatial-Spectral Denoising Framework for Robust Electrically Evoked Compound Action Potential Enhancement and Auditory Parameter Estimation

**DOI:** 10.3390/s25113523

**Published:** 2025-06-03

**Authors:** Fan-Jie Kung

**Affiliations:** Department of Electrical Engineering, National Taipei University of Technology, Taipei 10608, Taiwan; fjkung@mail.ntut.edu.tw

**Keywords:** electrically evoked compound action potential (ECAP), panoramic ECAP (PECAP), cochlear implant (CI), two-stage preprocessing denoising algorithm (TSPD), log-spectral amplitude (LSA), root mean square error (RMSE), two-dimensional correlation coefficient (TDCC), structural similarity index (SSIM), convolutional neural network (CNN)

## Abstract

The electrically evoked compound action potential (ECAP) is a crucial physiological signal used by clinicians to evaluate auditory nerve functionality. Clean ECAP recordings help to accurately estimate auditory neural activity patterns and ECAP magnitudes, particularly through the panoramic ECAP (PECAP) framework. However, noise—especially in low-signal-to-noise ratio (SNR) conditions—can lead to significant errors in parameter estimation. This study proposes a two-stage preprocessing denoising (TSPD) algorithm to address this issue and enhance ECAP signals. First, an ECAP matrix is constructed using the forward-masking technique, representing the signal as a two-dimensional image. This matrix undergoes spatial noise reduction via an improved spatial median (I-Median) filter. In the second stage, the denoised matrix is vectorized and further processed using a log-spectral amplitude (LSA) Wiener filter for spectral domain denoising. The enhanced vector is then reconstructed into the ECAP matrix for parameter estimation using PECAP. The above integrated spatial-spectral denoising framework is denoted as PECAP-TSPD in this work. Evaluations are conducted using a simulation-based ECAP model mixed with simulated and experimental noise, designed to emulate the spatial characteristics of real ECAPs. Three objective quality measures—namely, normalized root mean square error (RMSE), two-dimensional correlation coefficient (TDCC), and structural similarity index (SSIM)—are used. Simulated and experimental results show that the proposed PECAP-TSPD method has the lowest average RMSE of PECAP magnitudes (1.952%) and auditory neural patterns (1.407%), highest average TDCC (0.9988), and average SSIM (0.9931) compared to PECAP (6.446%, 5.703%, 0.9859, 0.8997), PECAP with convolutional neural network (CNN)-based denoising mask (PECAP-CNN) (9.700%, 7.111%, 0.9766, 0.8832), and PECAP with improved median filtering (PECAP-I-Median) (4.515%, 3.321%, 0.9949, 0.9470) under impulse noise conditions.

## 1. Introduction

The electrically evoked compound action potential (ECAP) is the combined response of auditory nerve fibers to electrical stimulation by a cochlear implant (CI) [1,2]. The ECAP is a critical signal for clinicians to evaluate the functionality of a patient’s auditory nerve fibers after CI surgery [3,4]. In the absence of feedback from CI users, the ECAP model can be of great value to clinicians in terms of assessing the hearing performance of CI users [5,6]. ECAP magnitude can be computerized and estimated using temporal or spatial methods [7,8,9]. The ECAP matrix can be constructed by measuring the ECAPs generated by the different electrode positions of the masker and probe stimuli. Such a method is referred to as the forward-masking technique [10]. Regarding the panoramic ECAP (PECAP) method [11,12], it has been shown that using a clean ECAP matrix can approximate auditory activity patterns and ECAP magnitudes [11,12], further assisting clinicians in evaluating a patient’s speech perception. Nevertheless, noisy ECAP matrices—particularly when the signal-to-noise ratio (SNR) is less than 10 dB—can introduce inaccuracies in estimating auditory activity patterns and ECAP magnitudes [11,12].

The abovementioned issue necessitates the implementation of noise reduction techniques on ECAP matrices before estimating the auditory activity patterns and ECAP magnitudes. These techniques can be classified into three primary categories: spatial filtering, temporal filtering, and spectral filtering. In spatial filtering, the mean and median filters represent well-established examples of spatial filtering [13,14,15]. The mean filter is a linear estimator employed to mitigate the adverse effects of image noise by eliminating random fluctuations. The principal disadvantage of the mean filter is that it results in image blurring, particularly at low SNRs. The median filter, which demonstrates superior noise removal performance compared to the mean filter, is a nonlinear estimator [16]. The median filter’s masking size tends to increase with elevated noise levels, which can result in the loss of information from the original images. To alleviate image distortion and achieve moderate noise reduction, an improved median filtering algorithm (I-Median) [17] has been developed, combining the advantages of mean and median filters to achieve superior denoising in adverse environments. Specifically, if the current pixel value is less than the average of the mask, the pixel value is replaced with the median of the mask. Otherwise, the pixel value keeps its original value [17].

In the field of time-domain signal analysis, adaptive filtering (AF) can be used to minimize noise. The AF method primarily uses algorithms such as least mean square (LMS) [18] and recursive least squares (RLS) [19] to iteratively adjust the weights, thereby reducing the adverse effects of noise on the signal. The advantage of AF methods is their near real-time implementation [20]. However, a critical aspect of these methods lies in the reference signal source, as clean signals—such as speech—are almost nonexistent in specific real-world scenarios. In addition, the choice of step size and filter order directly affects the error convergence rate, which is an important issue to address [21]. Finding the right balance is essential for noise reduction when AF is used.

Wiener filtering is an effective noise reduction technique in spectral domain signal analysis [22,23]. It estimates the noise’s power spectral density (PSD) to enhance the signal. However, at low SNRs, the accuracy of the estimated noise PSD may decline, directly impacting the noise reduction performance. To address this issue, a Wiener-based noise reduction algorithm known as log-spectral amplitude (LSA) Wiener filtering [24,25,26] has been developed to minimize the mean square error of the logarithmic spectrum. This approach helps to alleviate musical noise and improve the quality of speech signals under low-SNR conditions.

Deep learning techniques have recently been applied in the context of speech enhancement [27,28,29,30]. One such technique is the end-to-end convolutional recurrent neural network (CRN) [31], which enables near real-time implementation using a single microphone. This CRN algorithm can suppress noise under adverse conditions, such as a −5 dB SNR. However, the main drawback of these learning-based approaches is the substantial amount of data acquisition and preprocessing required [27,28,32], which can be both time- and resource-intensive. Because deep learning techniques are based on nonlinear approaches, the resulting distortion may be greater than that of the aforementioned linear approaches. Additionally, the noise reduction performance may degrade when encountering unseen scenarios.

Given the compromised estimation of auditory neural activity patterns and ECAP magnitudes when using PECAP under low-SNR conditions, this work proposes a method that combines PECAP with a two-stage preprocessing denoising algorithm (TSPD), referred to as PECAP-TSPD. Specifically, the ECAP signals are first denoised via TSPD and then used to estimate the neural parameters via PECAP. In the first stage of TSPD, the I-Median algorithm is used to reduce the random noise of an ECAP matrix, since the ECAP matrix can be considered an image. In the second step of TSPD, the denoised ECAP matrix is expanded and rearranged as a 1×NN vector, where N is the total number of electrodes. The rearranged vector can then be used as the input of the one-dimensional signal to LSA Wiener filtering for residual noise reduction. The PECAP-TSPD algorithm improves the accuracy of the estimates for key parameters, such as neural health and current spread. Simulated and measured noise are mixed into clean ECAP matrices to emulate the real ECAPs. This method can potentially assist in neural diagnosis, which warrants further validation using clinical ECAP data in future studies. The normalized root mean square error (RMSE), two-dimensional correlation coefficient (TDCC) [33], and structural similarity index (SSIM) [34,35] are employed as objective measures in this work to evaluate the performance of the unprocessed ECAP, processed ECAP signals by PECAP, PECAP with convolutional neural network (CNN)-based denoising mask (PECAP-CNN), PECAP with I-Median filtering (PECAP-I-Median), and PECAP-TSPD under various SNR conditions, noise densities, and scenarios.

The main contributions and insights of this study are as follows:

1. This study proposes a novel two-stage framework that combines spatial and spectral filtering for noise reduction in ECAP matrix signals.
2. A reordering technique is introduced to estimate the noise in the ECAP matrix, based on the physiological ECAP measurements obtained using the forward-masking technique [10].
3. A three-convolutional-layer neural network is proposed for denoising mask estimation. This network serves as one of the baselines, and is used to validate the noise estimation effect achieved by the proposed reordering technique.
4. The reordering ECAP vector is treated as a speech-like signal and further denoised in the second stage using LSA Wiener filtering.

The remainder of this article is organized as follows. Section 2 introduces the panoramic ECAP method. Section 3 describes the proposed method. Section 4 explains the settings and results. Section 5 concludes the study and outlines future work.

## 2. Panoramic ECAP Method

**Figure 1:**
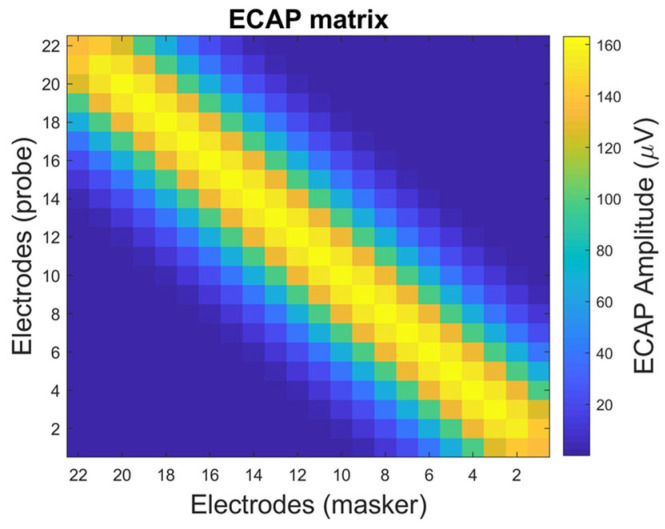
Illustration of an ECAP matrix simulation. The x-axis represents the masker electrode position (from Electrode 22 to Electrode 1), and the y-axis represents the prob electrode position (from Electrode 1 to Electrode 22) [12].

**Figure 2:**
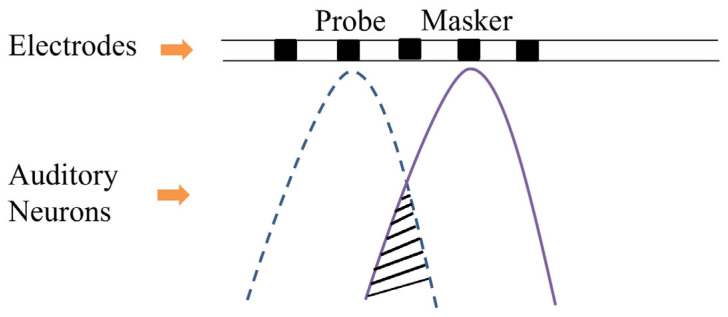
Schematic illustration of the overlapping area stimulated by the probe and the masker. Gaussian distributions represent the auditory neuron responses, and the shaded overlapping region indicates the ECAP response [6].

The panoramic ECAP (PECAP) method consists of two procedures: the forward-masking method and neural parameter estimation using sequential quadratic programming (SQP) [10,11,12]. The forward-masking method uses a probe and a masker to stimulate auditory neurons, generating the ECAP signal and reducing artifacts. This process constructs a matrix in which each element represents the measured ECAP signal from each pair of probe and masker positions, as illustrated in Figure 1. In Figure 1, the x-axis represents the position of the masker, and the y-axis represents the probe’s position. Figure 1 shows the simulated ECAP signal, illustrating the inverse relationship between the amplitude of the ECAP and the distance from the probe to the masker. When the positions of the probe and the masker are closer, the amplitude of the ECAP signal is larger. Conversely, when the probe and masker are farther apart, the amplitude of the ECAP signal is smaller. This phenomenon is explained in Figure 2 [6], where the neural activity pattern is assumed to follow a Gaussian distribution. The ECAP signal can be regarded as the overlapping area between two Gaussian distributions, corresponding to the neural activity patterns stimulated by the probe and masker. The overlap is maximized when the probe and the masker positions coincide. In contrast, the overlap is minimal when the probe and the masker positions are distant.

The auditory neural activity pattern is assumed to be a Gaussian distribution, defined as follows:

(1) $$a_{i}\left(k\right)=\alpha_{i}\eta_{i}exp\left\{-\frac{{\left(k-\mu_{i}\right)}^{2}}{2\sigma_{i}^{2}}\right\}$$

where i denotes the i-th electrode; k is the position along the cochlea; $\alpha_{i}$ and $\eta_{i}$ are the auditory neuron amplitude and neural health of the i-th electrode, respectively; $exp\left\{\cdot \right\}$ is an exponential function; $\mu_{i}$ and $\sigma_{i}$ represent the mean and standard deviation (also referred to as current spread [10] in physiological signals) of the Gaussian pattern for the i-th electrode, respectively. In this work, $\mu_{i}=i$ and $\alpha_{i}$ is prior information. Under the assumption that the ECAP signal is the overlap between the two auditory neuron responses of the probe and masker stimuli, the ECAP signal can be formulated as

(2) $$M\left(p,m\right)=\sum_{k=1}^{K}a_{p}\left(k\right)a_{m}\left(k\right),$$

where p is the index indicating that the p-th electrode is stimulated by the probe, m is the index indicating that the m-th electrode is stimulated by the masker, and K is set to N (the total number of electrodes) in this work. Equation (2) can also be expressed in matrix form as a clean ECAP matrix [12]:

(3) $$M=\sqrt{AA^{T}},$$

in which $A={\left[\begin{array}{ccc} a_{1} & a_{2} & \begin{array}{cc} \cdots & a_{N} \end{array} \end{array}\right]}^{T}\in R^{N\times K}$ is an auditory neural activity pattern matrix for each auditory neuron vector $a_{i}={\left[\begin{array}{ccc} a_{i}(1) & a_{i}(2) & \begin{array}{cc} \cdots & a_{i}(K) \end{array} \end{array}\right]}^{T}\in R^{K}$. Because A is a symmetric matrix, the matrix of the ECAP signal in Equation (3) is also a symmetric matrix. The measured ECAP matrix can be represented as

(4) $$M_{s}=M+U,$$

where U denotes a matrix that contains noise.

**Figure 3:**
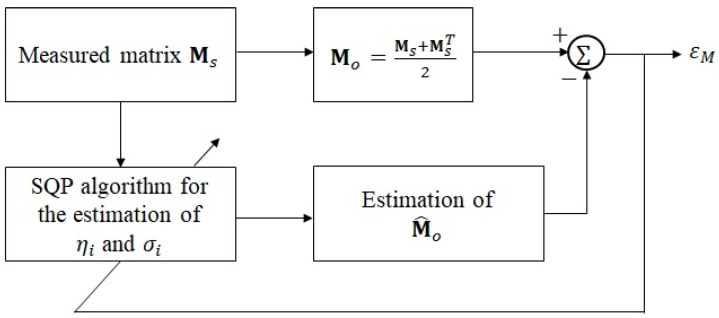
Block diagram of neural parameter estimation in the ECAP matrix using the SQP algorithm [6,12].

The random noise part can be alleviated using the following equation:

(5) $$M_{o}=\frac{M_{s}+M_{s}^{T}}{2},$$

where $M_{o}$ represents the denoised matrix of the ECAP signal. To further estimate $\eta_{i}$ and $\sigma_{i}$, the SQP, a large-scale constrained optimization algorithm, is utilized. Computerization of the parameter estimation of the ECAP matrix is illustrated in Figure 3.

In Figure 3, $\varepsilon_{M}$, which denotes the root mean square error between $M_{o}$ and estimated denoised ECAP matrix ${\hat{M}}_{o}$, is formulated as

(6) $$\varepsilon_{M}=\sqrt{{\frac{1}{N^{2}}\left|M_{o}\left(p,m\right)-{\hat{M}}_{o}\left(p,m\right)\right|}^{2}},$$

where $M_{o}(p,m)$ and ${\hat{M}}_{o}(p,m)$ represent the (p,m)-th entry of $M_{o}$ and ${\hat{M}}_{o}$, respectively. In this work, different SNR and density conditions are also introduced to assess the parameter estimation capability using SQP.

**Figure 4:**
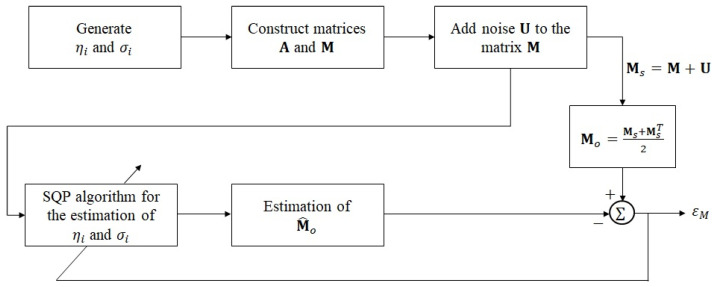
Block diagram of parameter estimation under additive noise scenarios using the SQP algorithm [11,12].

Figure 4 depicts a structure designed to test the noise resistance performance using the SQP algorithm. The above procedures can eliminate most of the noise. However, in low-SNR scenarios, the distortion increases due to the error estimation of $\eta_{i}$ and $\sigma_{i}$. Therefore, the preprocessing algorithm is developed below.

## 3. Proposed Method

The proposed method involves two stages for noise reduction. The first stage of the denoising process is described below.

### 3.1. First Stage of Noise Reduction Processing

In light of the detrimental effect of noise on the estimation of neural parameters, this work proposes a two-stage preprocessing denoising (TSPD) algorithm for the ECAP matrix before the PECAP algorithm. First, the ECAP matrix is treated as an image. In the first stage of TSPD, the improved median (I-Median) filtering algorithm is applied to reduce noise, as shown in Equation (7).

(7) $$M_{I}\left(p,m\right)=\left\{\begin{array}{cc} Median\left(p,m\right) & M\left(p,m\right)<Avg(p,m)\\ M\left(p,m\right) & otherwise \end{array}\right.,$$

where Median(p,m) and Avg(p,m) denote the processed values using the median and mean filters at positions (p,m), respectively. $M_{I}(p,m)$ denotes the processed result obtained using I-Median filtering. This work sets the kernel sizes of median and mean filtering to 3×3. Equation (7) describes that if the ECAP value at positions (p,m) is less than the mean filter processing value, the ECAP value is considered noise and can be replaced by the median filter processing value. Conversely, if the ECAP value is greater, it is retained. The processed ECAP matrix is expressed as

(8) $$M_{I}=\left[\begin{array}{ccc} M_{I}(1,1) & \ldots & M_{I}(1,N)\\ \vdots & \ddots & \vdots \\ M_{I}(N,1) & \ldots & M_{I}(N,N) \end{array}\right].$$

The I-Median algorithm is suitable for removing low-to-medium-density noise from an image. In some cases, however, the noise is distributed across all pixels of an image. To further deal with this noise, LSA Wiener filtering is employed after I-median filtering by expanding $M_{I}$ into a 1×NN vector. The following reordering procedure is important because LAS Wiener filtering uses the first few time frames as the noise component to estimate the initial noise PSD to reduce noise recursively. Therefore, selecting which elements of the ECAP matrix have high probabilities of being noise is a critical step. The following reordering rule not only expands $M_{I}$ into a 1×NN vector, but also selects which elements of $M_{I}$ are most likely noise based on the physiological characteristic of the ECAP matrix, as described in Section 2. The reordering rule for transforming the ECAP matrix into the ECAP vector is as follows:

1. Calculate the absolute value of index p minus index m.
  (9) $$B=\left[\begin{array}{ccc} \left|1-1\right| & \ldots & \left|1-N\right|\\ \vdots & \ddots & \vdots \\ \left|N-1\right| & \ldots & \left|N-N\right| \end{array}\right]=\left[\begin{array}{ccc} 0 & \ldots & \left|1-N\right|\\ \vdots & \ddots & \vdots \\ \left|N-1\right| & \ldots & 0 \end{array}\right],$$
  where B is an index matrix that records the absolute value of the position difference between p and m.
2. Record |p−m| for each element in B and concatenate each row into a long vector.
  (10) $$b=\left[b\left(1,1\right),\ldots ,b\left(1,N\right),b\left(2,1\right),\ldots ,b\left(2,N\right),\ldots ,b\left(N,1\right),\ldots ,b(N,N)\right]\in R^{1\times NN},$$
  where $b\left(p,m\right)=\left|p-m\right|$ is the (p,m)-th entry of B.
3. Order b in descending order and record the descending order index.
  (11) $$\left(i_{d},\bar{b}\right)=descend(b),$$
  where $descend\left(\cdot \right)$ represents the descending order operation, $\bar{b}$ is a vector based on the descending order of the results of b, and $i_{d}$ is the index vector corresponding to $\bar{b}$.
4. The desired vector can then be obtained using the following equation:
  (12) $$m_{I}=\left[M_{I}\left(i_{d}\left(1\right)\right),M_{I}\left(i_{d}\left(2\right)\right),\ldots ,M_{I}\left(i_{d}\left(NN\right)\right)\right]\in R^{1\times NN},$$
  where $m_{I}$ is a 1×NN row vector containing both noise components and noisy ECAP signal components. The noise, where the ECAP signal is weak, is placed in the first part of the vector, while the target signal, where the ECAP signal is stronger, is placed in the last part of the vector. The reordering process is part of the second stage of TSPD, which employs LSA Wiener filtering for improved noise reduction, as stated below.

### 3.2. Second Stage of Noise Reduction Processing

In the second stage of TSPD, LSA Wiener filtering is utilized to address the residual noise in the vector $m_{I}$, which is treated as a discrete-time series input. The first step is to transform $m_{I}$ into a short-time Fourier transform (STFT) domain, as shown in Equation (13).

(13) $$Y_{m}\left(l,k\right)={F\left\{m_{I}(n+lN_{hop})w(n)\right\}}_{zero-padded\;to\;2N_{w}},$$

where $F\left\{\cdot \right\}$ denotes the fast Fourier transform (FFT) operator. $Y_{m}\left(l,k\right)$ is the STFT signal of $m_{I}$. l and k denote the time frame and frequency indices, respectively. $N_{hop}$ is the hop size. w(n) is the window of length $N_{w}$ for short-time signal analysis. The signal segment is zero-padded to a length of ${2N}_{w}$ to ensure adequate frequency resolution without aliasing. The LSA Wiener filter aims at minimizing the log-spectral amplitude

(14) $$J=arg\underset{H(l,k)}{\min}E\left\{{\left|\log_{e}\left|X\left(l,k\right)\right|-\log_{e}\left|\hat{X}\left(l,k\right)\right|\right|}^{2}\right\},$$

where J is the cost function to minimize the mean square error of log-spectral amplitudes $\log_{e}\left|X\left(l,k\right)\right|$ and $\log_{e}\left|\hat{X}\left(l,k\right)\right|$. $X\left(l,k\right)$ and $X\left(l,k\right)$ are the clean signal and estimated clean signal spectra, respectively. The optimal solution is [24,25,26]

(15) $$H_{LSA}\left(l,k\right)=\frac{\xi (l,k)}{1+\xi (l,k)}exp\left\{\frac{1}{2}\int_{\nu (l,k)}^{\infty }\frac{e^{-t}}{t}dt\right\},$$

where $\xi \left(l,k\right)=E\left\{{\left|X(l,k)\right|}^{2}\right\}/P_{u}\left(l,k\right)$ is the prior SNR with $P_{x}\left(l,k\right)=E\left\{{\left|X(l,k)\right|}^{2}\right\}$ being a clean signal PSD and $P_{u}\left(l,k\right)=E\left\{{\left|U_{r}(l,k)\right|}^{2}\right\}$ being a residual noise PSD. $\upsilon \left(l,k\right)=\left(\xi \left(l,k\right)/1+\xi \left(l,k\right)\right)\gamma (l,k)$ with $\gamma \left(l,k\right)={\left|Y_{m}(l,k)\right|}^{2}/P_{u}\left(l,k\right)$ representing the posterior SNR. $\xi \left(l,k\right)$ can be estimated using the decision-directed approach as described below:

(16) $$\hat{\xi }\left(l,k\right)=\alpha \frac{{\left|X(l-1,k)\right|}^{2}}{P_{u}\left(l-1,k\right)}+(1-\alpha )max\left\{\gamma \left(l,k\right)-1,0\right\},$$

where α is a forgetting factor. $P_{u}\left(l,k\right)$ can be estimated and updated using the following log-likelihood ratio criterion:

(17) $$\Lambda \left(l,k\right)=ln\frac{f(Y_{m}(l,k)|H_{1})}{f(Y_{m}(l,k)|H_{0})}=-\ln\left(1+\xi \left(l,k\right)\right)+\upsilon (l,k),$$

where

(18) $$f\left(Y_{m}\left(l,k\right)|H_{1}\right)=\frac{1}{\pi ({P_{x}\left(l,k\right)+P}_{u}\left(l,k\right))}exp\left\{-\frac{{\left|Y_{m}(l,k)\right|}^{2}}{P_{x}\left(l,k\right)+P_{u}\left(l,k\right)}\right\}$$

is the conditional probability density function (PDF) of $Y_{m}\left(l,k\right)$ given that the event ($H_{1}$) of the ECAP signal occurs.

(19) $$f\left(Y_{m}\left(l,k\right)|H_{0}\right)=\frac{1}{\pi P_{u}\left(l,k\right)}exp\left\{-\frac{{\left|Y_{m}(l,k)\right|}^{2}}{P_{u}\left(l,k\right)}\right\}$$

is the conditional PDF of $Y_{m}\left(l,k\right)$ assuming only noise occurs at event $H_{0}$. $\upsilon \left(l,k\right)=\gamma \left(l,k\right)\xi \left(l,k\right)/(1+\xi \left(l,k\right))$. If $\sum_{l=1}^{N_{t}}\Lambda \left(l,k\right)$ is less than a small value $\varepsilon_{1}$, the noise PSD can be updated using the following recursive averaging.

(20) $${\hat{P}}_{u}\left(l,k\right)=\alpha {\hat{P}}_{u}\left(l-1,k\right)+\left(1-\alpha \right){\left|Y_{m}\left(l,k\right)\right|}^{2}.$$

The estimated clean ECAP signal can be obtained using the following equation:

(21) $$\hat{X}\left(l,k\right)=H_{LSA}\left(l,k\right)Y_{m}(l,k).$$

The complete LAS Wiener filtering procedures used in the second noise reduction stage are listed in Table 1.

In Table 1, $N_{u}$ is the number of time frames used to estimate the initial noise PSD. Next, the processed row vector $\hat{X}\left(l,k\right)$ is converted into the inverse STFT, the time domain signal $\hat{x}\left(n\right)$, which can be reconstructed into the matrix format, as described below:

(22) $${\tilde{M}}_{I}=\left[\begin{array}{ccc} \hat{x}(n^{\prime }|i_{d}\left(n^{\prime }\right)=1) & \ldots & \hat{x}(n^{\prime }|i_{d}\left(n^{\prime }\right)=N)\\ \begin{array}{c} \hat{x}(n^{\prime }|i_{d}\left(n^{\prime }\right)=N+1)\\ \vdots \end{array} & \ddots & \begin{array}{c} \hat{x}(n^{\prime }|i_{d}\left(n^{\prime }\right)=2N)\\ \vdots \end{array}\\ \hat{x}(n^{\prime }|i_{d}\left(n^{\prime }\right)=\left(N-1\right)N+1) & \ldots & \hat{x}(n^{\prime }|i_{d}\left(n^{\prime }\right)=NN) \end{array}\right].$$

where $i_{d}\left(n^{\prime }\right)$ is defined in Equation (11), with $n^{\prime }$ being a range from 1 to NN. In this work, the mean filter is applied in ${\tilde{M}}_{I}$ if the SNR value is below 4 dB, to leverage the advantage of random noise removal at low SNRs [15]. The simulation arrangement, experimental settings, and results are provided in Section 4.

## 4. Settings and Results

The simulation arrangement and results are described below.

### 4.1. Simulation Arrangement and Results

**Figure 5:**
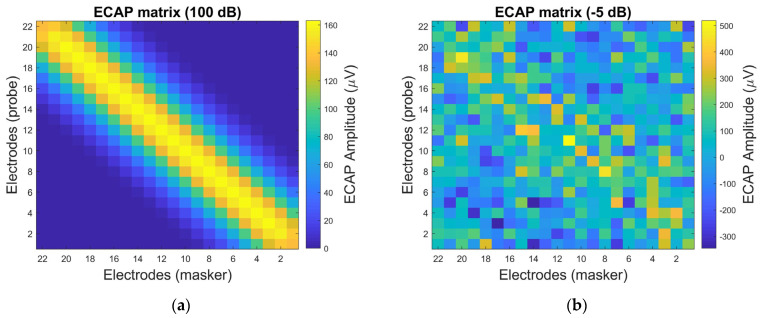

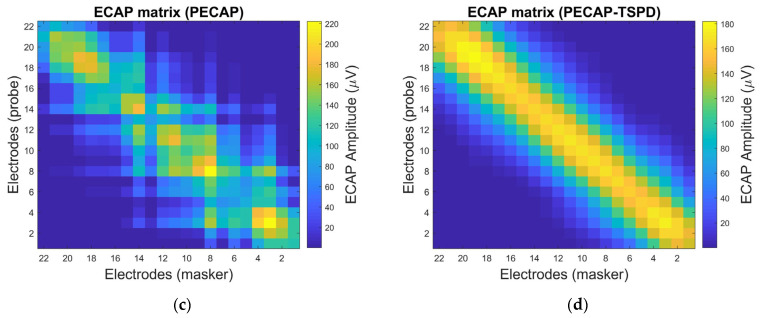
Results of (**a**) the ECAP matrix at 100 dB SNR, (**b**) the noisy ECAP matrix at −5 dB SNR, (**c**) the ECAP matrix processed using the PECAP method at −5 dB SNR, and (**d**) the ECAP matrix processed using the TSPD method at −5 dB. The neural health and current spread settings correspond to Scenario 1 listed in Table 2.

Two types of noise—random noise and impulse noise—are used to evaluate the performance of the proposed PECAP-TSPD algorithm. Twelve SNR levels (−5 dB, −2 dB, 1 dB, 4 dB, 7 dB, 10 dB, 13 dB, 16 dB, 19 dB, 22 dB, 25 dB, and 100 dB—representing the clean ECAP signal situation for the random noise case) are used in the random noise. Four densities—10%, 20%, 30%, and 40%—are used in the impulse noise. The normalized RMSE is used as an objective quality measure to calculate the error between the ground truth and the estimated results (auditory neural activity pattern and ECAP amplitude). The two-dimensional correlation coefficient (TDCC) [33] and structural similarity index (SSIM) [34,35] are used to evaluate the similarity between the clean ECAP matrix and the reconstructed ECAP matrix. The STFT parameter settings in this work are as follows: A rectangular window with a length of 22 is used. The FFT size is 44. No overlap for each segment ($N_{hop}=N_{w}$). In Table 1, $N_{u}=6$, α=0.96, and $\varepsilon_{1}=0.15$. Seven different combinations of neural health and current spread are listed in Table 2. The results of the ECAP matrices before and after processing at −5 dB of SNR are shown in Figure 5. The simulation software used in this study is MATLAB 2018b.

In Figure 5b, the ECAP matrix is filled with random noise, making it challenging to observe the pristine measured ECAP data, as depicted in Figure 5a. The PECAP and PECAP-TSPD methods can mitigate the detrimental effects of boisterous environments to recover the clean ECAP matrix shown in Figure 5c,d. The TDCC results for the noisy ECAP matrix, the PECAP matrix using PECAP, and the PECAP matrix using PECAP-TSPD are 0.4049, 0.8950, and 0.9960, respectively. Meanwhile, the SSIM results for these matrices are 0.0723 for the noisy ECAP matrix, 0.6526 for the processed PECAP matrix using PECAP, and 0.9681 for the processed PECAP matrix using PECAP-TSPD. The results of TDCC and SSIM are greater than 0.96, further indicating the satisfactory performance of PECAP-TSPD under adverse noisy conditions.

**Figure 6:**
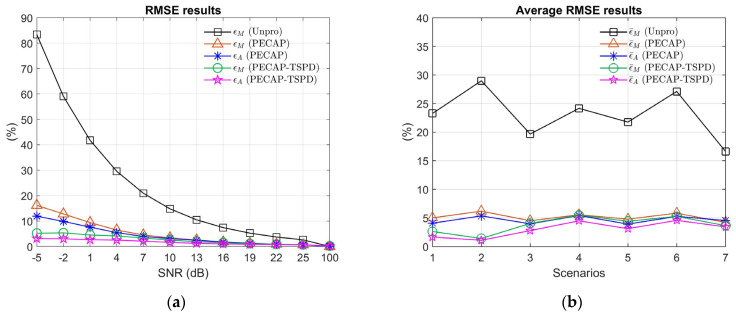
Normalized RMSE and average normalized RMSE of ECAP magnitudes and auditory neural activity patterns under twelve SNR conditions: (**a**) in Scenario 1 and (**b**) across Scenarios 1 to 7. The unprocessed ECAP matrices are compared with the baseline PECAP and the proposed PECAP−TSPD methods. Parameter settings for each scenario are provided in Table 2.

The results of the normalized RMSE of the ECAP magnitude ($\epsilon_{M}$) and auditory neural activity pattern ($\epsilon_{A}$) are depicted in Figure 6.

(23) $$\epsilon_{M}=\frac{\varepsilon_{M}}{{\bar{M}}_{o}},$$

where ${\bar{M}}_{o}$ is the maximum absolute value of $M_{o}$. $\epsilon_{A}$ is computed as

(24) $$\epsilon_{A}=\sqrt{{\frac{1}{N^{2}}\left|A\left(p,m\right)-\hat{A}\left(p,m\right)\right|}^{2}}/\bar{A},$$

where $\bar{A}$ is the maximum absolute value of A. A(p,m) and $\hat{A}(p,m)$ denote the (p,m)-th entry of A and $\hat{A}$, respectively. $\hat{A}$ is the estimated auditory neural activity pattern. Figure 6a shows the normalized RMSE of the unprocessed ECAP signals, ECAP signals processed by PECAP, and PECAP-TSPD algorithms for different SNRs in Scenario 1. The normalized RMSE of the magnitude of the unprocessed ECAP signals at −5 dB SNR increases to 83.39%, which is comparably higher than those of the processed ECAP signals by PECAP (16.17%) and PECAP-TSPD (5.23%), indicating the need for ECAP signal processing. When comparing the $\epsilon_{A}$ between PECAP and PECAP-TSPD processing ECAP signals, the values of $\epsilon_{A}$ by PECAP-TSPD are all smaller than those by PECAP, except the 100 dB SNR case, where $\epsilon_{A}$ are 0.0043% and 0.1885% for PECAP and PECAP-TSPD, respectively. The RMSE results of $\epsilon_{M}$ for PECAP and PECAP-TSPD under 16, 19, 22, and 25 SNRs are shown in Table 3. Table 3 shows that the PECAP-TSPD algorithm decreases the RMSE when the SNR values are below 25 dB. The difference in RMSE remains approximately the same when the SNR is increased to 22 dB. The above results indicate that it is unnecessary to use TSPD before PECAP when the SNR is 25 dB or higher. Figure 6b shows the curve of the average normalized RMSE, denoted as ${\bar{\epsilon }}_{M}$ and ${\bar{\epsilon }}_{A}$ for the ECAP magnitude and auditory neural activity, respectively. Compared to the unprocessed and processed ECAP signals, the maximum values of average normalized RMSE are 6.17% and 5.48% for PECAP and PECAP-TSPD, respectively. In contrast, the maximum value of the unprocessed ECAP signals is 28.97%, showing the noise resistance capabilities of the PECAP and PECAP-TSPD algorithms. The performance of the PECAP-TSPD algorithm is superior to that of the PECAP algorithm, as the values of ${\bar{\epsilon }}_{M}$ and ${\bar{\epsilon }}_{A}$ obtained with PECAP-TSPD are lower than those obtained with PECAP. The average of ${\bar{\epsilon }}_{M}$ from Scenarios 1 to 7 can be ranked as PECAP-TSPD (3.83%), PECAP (5.14%), and unprocessed (23.07%). The averages of ${\bar{\epsilon }}_{A}$ from Scenarios 1 to 7 for PECAP-TSPD and PECAP are 3.01% and 4.64%, respectively.

**Figure 7:**
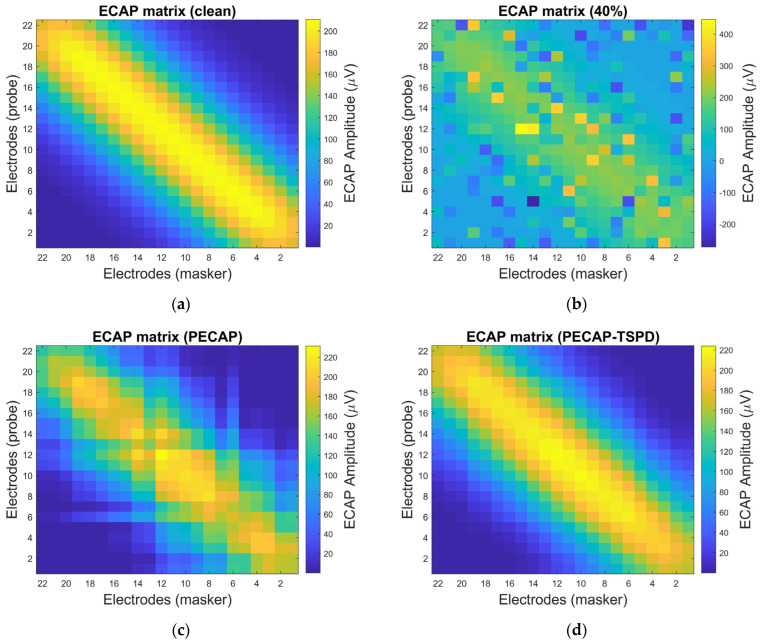
ECAP matrix results under impulse noise conditions: (**a**) no impulse noise, (**b**) impulse noise with 40% density, (**c**) ECAP matrix processed using the PECAP method under 40% density impulse noise, and (**d**) ECAP matrix processed using the PECAP−TSPD method under 40% density impulse noise. The neural health and current spread settings correspond to Scenario 2 in Table 2.

Next, the impulse noise is added to the EACP matrix to evaluate the performance of PECAP-TSPD under four different densities. Figure 7 illustrates the results of the ECAP matrices before and after using the PECAP and PECAP-TSPD algorithms at the 40% density of the impulse noise.

The impulse noise with 40% density heavily contaminates the original ECAP matrix (Figure 7a), as shown in Figure 7b, emphasizing the importance of signal processing. The ECAP matrices processed using the PECAP and PECAP-TSPD algorithms are depicted in Figure 7c,d, where the impulse noise is most reduced. When comparing Figure 7c with Figure 7d, the restored ECAP matrix in Figure 7d shows more resemblance to that in Figure 7a than to that in Figure 7c, indicating the satisfactory performance of PECAP-TSPD under adverse noisy environments.

**Figure 8:**
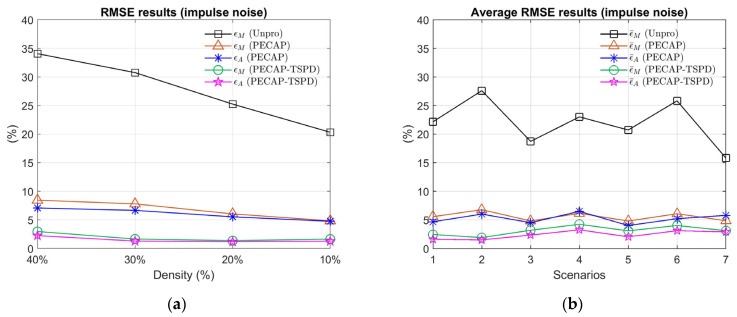
Normalized RMSE and average normalized RMSE of ECAP magnitudes and auditory neural activity patterns under four impulse noise densities: (**a**) in Scenario 2 and (**b**) across Scenarios 1 to 7. The unprocessed ECAP matrices are compared with the PECAP and PECAP-TSPD algorithms. The parameter settings for each scenario are listed in Table 2.

The normalized RMSE results of the ECAP magnitude and neural activity pattern of the impulse noise case are depicted in Figure 8.

Figure 8a presents the $\epsilon_{M}$ and $\epsilon_{A}$ curves at four distinct densities in Scenario 2 for the unprocessed, PECAP, and PECAP-TSPD approaches, all of which increase as the impulse noise density increases. In the case of 40% impulse noise density, the $\epsilon_{M}$ values for unprocessed, PECAP, and PECAP-TSPD are 34.07%, 8.44%, and 2.96%, respectively, displaying the effectiveness of PECAP and PECAP-TSPD in adverse noisy environments. The average normalized RMSE results are depicted in Figure 8b. The maximum values of ${\bar{\epsilon }}_{M}$ for the PECAP and PECAP-TSPD algorithms are 6.77% and 4.22%, respectively. For the unprocessed ECAP matrices, the maximum value of ${\bar{\epsilon }}_{M}$ is 27.59%, which suggests that PECAP and PECAP-TSPD are robust against noise. The PECAP-TSPD algorithm performs better than the PECAP algorithm because the values of ${\bar{\epsilon }}_{M}$ and ${\bar{\epsilon }}_{A}$ calculated by PECAP-TSPD are lower than those estimated by PECAP. The mean values of ${\bar{\epsilon }}_{M}$ from Scenarios 1 through 7 can be arranged in ascending order as follows: PECAP-TSPD (3.13%), PECAP (5.57%), and unprocessed (21.97%). The mean values of ${\bar{\epsilon }}_{A}$ from Scenarios 1 to 7 for PECAP-TSPD and PECAP are 2.39% and 5.23%, respectively. To validate the proposed reordering technique for noise region estimation of ECAP matrices, a three-convolutional-layer neural network for denoising mask estimation is proposed as follows.

### 4.2. CNN-Based Denoising Mask Estimation

**Figure 9:**
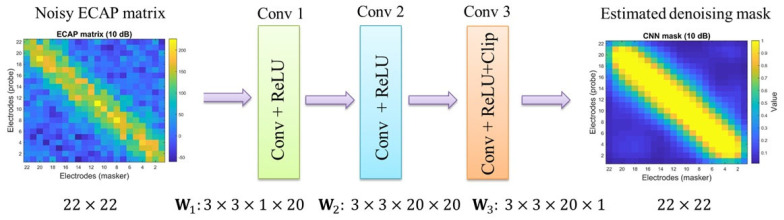
Schematic of the three−convolutional−layer denoising mask.

The schematic of the denoising mask estimation of the CNN-based network is presented in Figure 9.

The schematic of the proposed three-convolutional-layer neural network for the denoising mask estimation is inspired by [31,36]; that is, training the two-dimensional kernels as feature maps, which can match the image property, such as the ECAP matrix in this work. Second, learning a mask between 0 and 1 for the CNN-based structure is easier than training on the clean image. The sizes of the input and output data are 22×22. The three training weights are denoted as $W_{1}$, $W_{2}$, and $W_{3}$.

**Figure 10:**
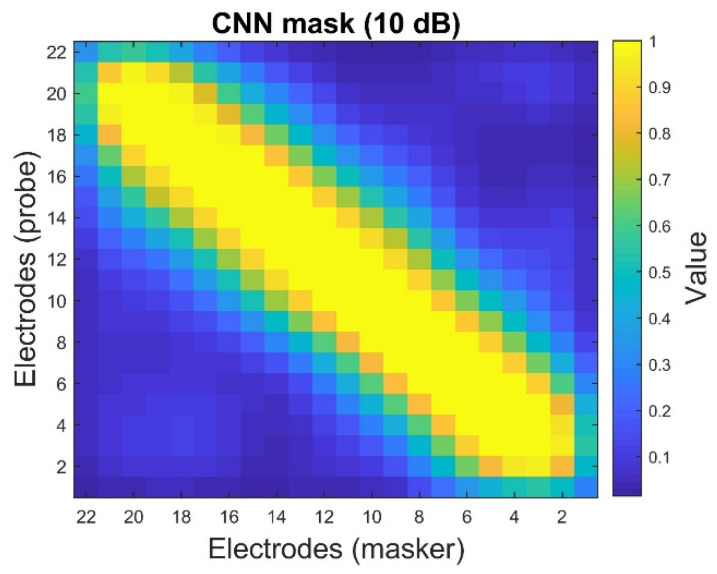
The convolutional neural network (CNN)-based denoising mask at 10 dB SNR. The mask values lie within the range from 0 to 1.

The kernel size is set to 3×3. The rectified linear unit (ReLU) is used as the activation function for each convolutional layer. The clip operator is used in the third convolutional layer to ensure the output value is between 0 and 1. The data size for each convolutional layer is stated in Table 4. The loss function is described in the following equation:

(25) $$l\left(\Theta \right)=\frac{1}{2N_{s}}\sum_{i=1}^{N_{s}}{\left\|x_{i}-Q⊚y_{i}\right\|}_{F}^{2},$$

where Θ denotes the hyperparameters. $N_{s}=700$ denotes the total number of training sample pairs in this work. $x_{i}$ is the *i*-th clean ECAP matrix and $y_{i}$ is the *i*-th noisy ECAP matrix. Q is the training denoising mask. ⊚ is the Hadamard product operator [37]. ${\left\|\cdot \right\|}_{F}$ is the Frobenius norm [38]. The results are shown in Figure 10.

**Figure 11:**
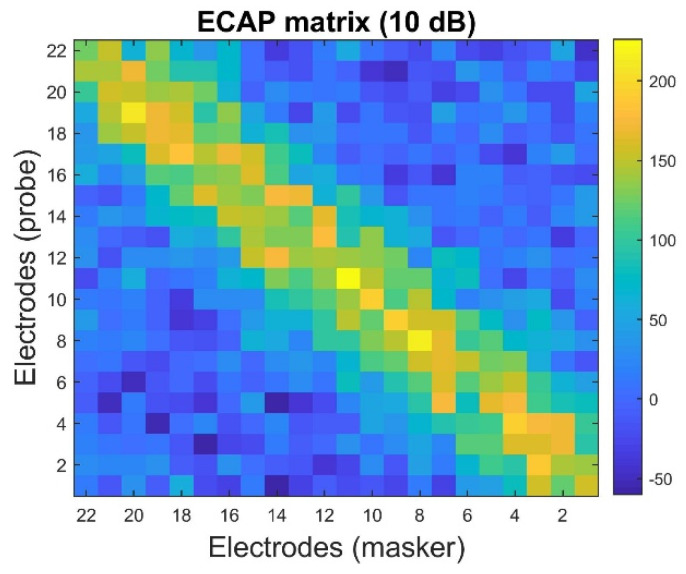
Results of the noisy ECAP matrix. The SNR is set to 10 dB. The neural health and current spread settings correspond to Scenario 1, as presented in Table 2.

Figure 10 shows that the values of the CNN mask are close to one when the probe and the mask’s positions approach the diagonal term (i.e., the same electrode position). The values of the CNN mask become smaller, even reaching zero, if the probe and the mask’s positions are distant. These indicate that the theoretical assumption above can be validated. The neural health and current spread settings in Figure 10 correspond to Scenario 1 in Table 2. The noisy ECAP matrix at 10 dB SNR under the same neural parameter settings is depicted in Figure 11.

The results of the clean ECAP matrix (Figure 5a) suggest that the CNN-based denoising mask (Figure 10) can approximately estimate the signal and noise components from the noisy ECAP matrix (illustrated in Figure 11). TDCC [33] and SSIM [34,35] are used to evaluate the similarity between the CNN-based denoising mask and the clean ECAP matrix, and compare it to that of the noisy ECAP matrix. TDCC and SSIM can be implemented using the *corr2* and *ssim* functions in MATLAB R2018b. The results are described below.

Table 5 shows that the estimated CNN mask is more similar to the clean ECAP matrix than the noisy ECAP matrix. The TDCC and SSIM results offer an empirical justification for the reordering procedure. The low-SNR components are distributed in the off-diagonal region of the noisy ECAP matrix. The high SNR components are distributed in the diagonal region of the noisy ECAP matrix. The above theoretical assumption and the empirical results explain why this study utilizes the descending order operator to select the maximum value of $\left|p-m\right|$. Then, the corresponding value of (p,m)-th position of the noisy ECAP matrix can be regarded as the noise to insert the first position of the reordering ECAP vector for further noise reduction using the LSA Wiener filter. The following section discusses the performance with and without LSA Wiener filtering after I-Median filtering.

### 4.3. LSA Wiener Filtering Improvements After I-Median Filtering

**Figure 12:**
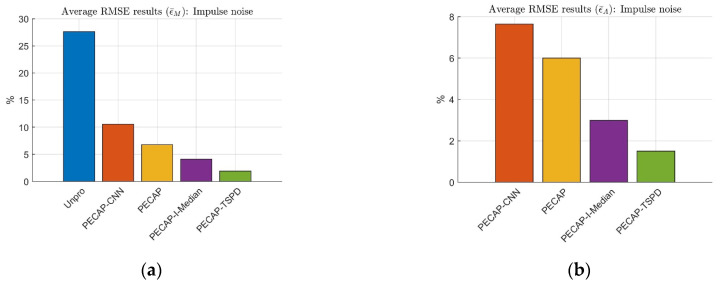
Average RMSE results of (**a**) ECAP matrix magnitude and (**b**) auditory neural activity pattern. Unprocessed ECAP data and ECAP data processed with PECAP-CNN, PECAP, PECAP-I-Median, and PECAP-TSPD are used. The clean ECAP matrix is mixed with impulse noise at densities of 10%, 20%, 30%, and 40%. The neural health and current spread settings correspond to Scenario 2, as presented in Table 2.

This work explains the importance of applying LSA Wiener filter processing after I-Median filtering in situations involving impulse noise. The results are shown in Figure 12.

In Figure 12, PECAP-CNN refers to the method that integrates the proposed CNN-based denoising mask with PECAP. The PECAP-I-Median method incorporates I-Median filtering into PECAP. In contrast, PECAP-TSPD combines the two-stage preprocessing denoising (TSPD) algorithm with PECAP. Figure 12a shows that the four preprocessing approaches can reduce the RMSE by 16% compared to the unprocessed ECAP data (Unpro). Although the CNN-based denoising mask can estimate the dominant signal and noise regions of the noisy ECAP matrix, directly multiplying the noisy ECAP matrix with the estimated CNN-based denoising mask produces more distortion than the other three preprocessing approaches. That is because the deep learning technique belongs to the nonlinear-based approach. PECAP-I-Median is slightly superior to PECAP, suggesting the effectiveness of the I-Median filtering under impulse noise conditions. PECAP-TSPD performs better than PECAP-I-Median, which indicates that the benefit of LSA Wiener filtering comes when used after the I-Median filtering. The ${\bar{\epsilon }}_{M}$ rank is as follows: PECAP-TSPD (1.905%), PECAP-I-Median (4.141%), PECAP (6.779%), PECAP-CNN (10.550%), and Unpro (27.590%). For the auditory neural activity pattern analysis in Figure 12b, the RMSE results of ${\bar{\epsilon }}_{A}$ can be ranked as PECAP-TSPD (1.503%), PECAP-I-Median (2.986%), PECAP (6.002%), and PECAP-CNN (7.638%). In addition, this work evaluated the mentioned preprocessing algorithms using TDCC and SSIM. The results are described in Table 6.

**Figure 13:**
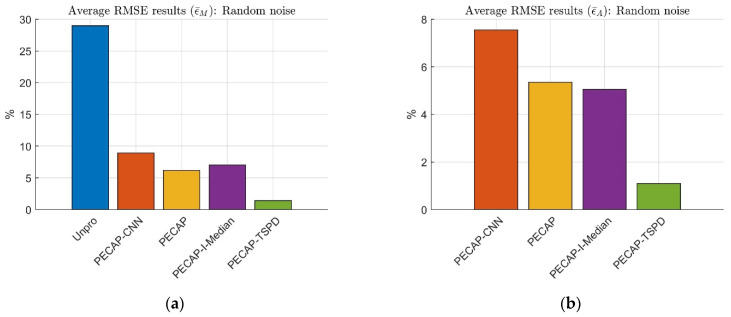
Average RMSE results of (**a**) ECAP matrix magnitude and (**b**) auditory neural activity pattern. Unprocessed ECAP data and ECAP data processed with PECAP-CNN, PECAP, PECAP-I-Median, and PECAP-TSPD are used. The clean ECAP matrix is combined with random noise at SNRs of −5 dB, −2 dB, 1 dB, 4 dB, 7 dB, 10 dB, 13 dB, 16 dB, 19 dB, 22 dB, 25 dB, and 100 dB. The neural health and current spread settings correspond to Scenario 2, as detailed in Table 2.

Table 6 shows the same trend as Figure 12; that is, the average TDCC and average SSIM rank performances are PECAP-TSPD, PECAP-I-Median, PECAP, and PECAP-CNN. The above results show that the proposed TSPD-PECAP algorithm performs better than PECAP-I-Median in impulse noise situations. This work evaluated the aforementioned preprocessing approaches under random noise conditions. The results are presented in Figure 13.

Figure 13 shows that PECAP and PECAP-I-Median have almost the same performance. For ECAP matrix magnitude analysis, the average RMSE of ${\bar{\epsilon }}_{M}$ for PECAP and PECAP-I-Median are 6.172% and 7.031%, respectively. For auditory neural activity pattern analysis, the average RMSE of ${\bar{\epsilon }}_{A}$ for PECAP and PECAP-I-Median are 5.347% and 5.055%, respectively. These results indicate that I-Median filtering has a limitation in dealing with various SNR random noise conditions. It emphasizes the necessity of LSA Wiener filtering. The ${\bar{\epsilon }}_{M}$ rank is as follows: PECAP-TSPD (1.400%), PECAP (6.172%), PECAP-I-Median (7.031%), PECAP-CNN (8.910%), and Unpro (28.970%). Similarly, the ${\bar{\epsilon }}_{A}$ rank is as follows: PECAP-TSPD (1.091%), PECAP-I-Median (5.055%), PECAP (5.347%), and PECAP-CNN (7.551%). TDCC and SSIM are also used to assess the performance of preprocessing approaches. The results of TDCC and SSIM are listed in Table 7.

The results in Table 7 show a similar trend to Figure 13, in that the rank performance in terms of average TDCC is PECAP-TSPD, PECAP-I-Median, PECAP, and PECAP-CNN. The rank performance in terms of average SSIM is PECAP-TSPD, PECAP, PECAP-I-Median, and PECAP-CNN. The proposed PECAP-TSPD algorithm performs robustly in random noise cases with various SNRs.

The window length affects the sensitivity of this proposed method; a shorter window length results in lower frequency resolution, whereas a longer length can lead to a decline in noise power spectral density (PSD) estimation accuracy. The results of the two-dimensional correlation coefficient (TDCC) [33] and the structural similarity index (SSIM) [34,35] with different window sizes are shown in Table 8.

The neural health and current spread settings in Table 8 align with Scenario 2, as detailed in Table 2. The TDCC and SSIM results for a window length of 22 are slightly better than those obtained with a window length of 11. However, when the window length increases to 44, the SSIM value drops significantly, from 0.9920 to 0.5521. Therefore, the window length is set to 22 in this work.

To emulate the real ECAP measurements, the clean ECAP matrix is mixed with measured noise as provided in the following section.

### 4.4. Experimental Results

**Figure 14:**
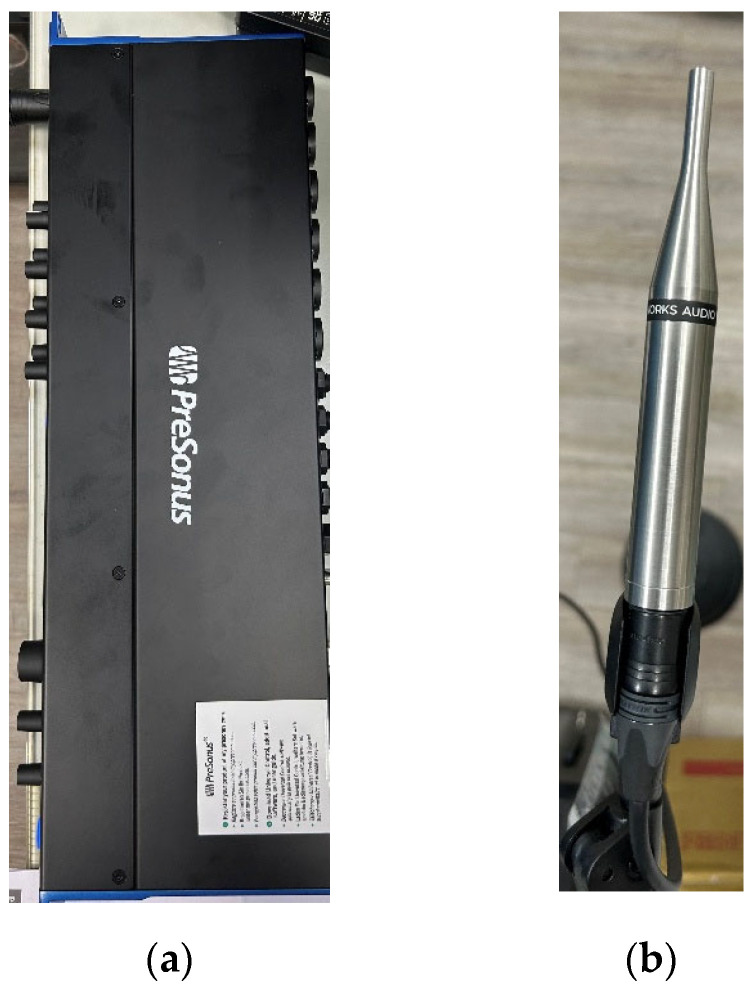
Experimental equipment of (**a**) PreSonus Studio 1824c audio interface and (**b**) Earthworks Audio M23 omnidirectional measurement microphone.

This work utilizes the PreSonus Studio 1824c audio interface and the Earthworks Audio M23 omnidirectional measurement microphone to record noise, emulating a real-world ECAP recording scenario. The experimental equipment is shown in Figure 14.

**Figure 15:**
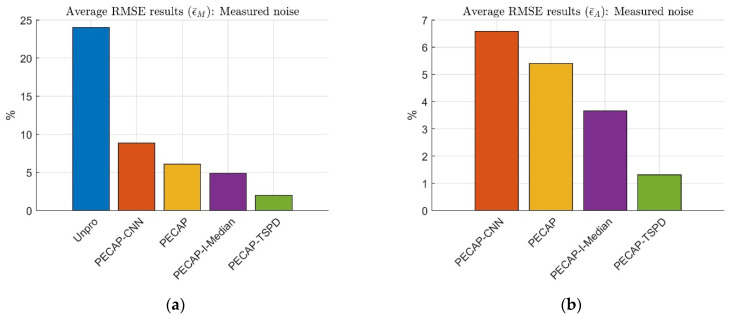
The average RMSE experimental results of (**a**) ECAP matrix magnitude and (**b**) auditory neural activity pattern. Both unprocessed ECAP data and ECAP data processed with PECAP-CNN, PECAP, PECAP-I-Median, and PECAP-TSPD are evaluated. The clean ECAP matrix is mixed with measured noise at densities of 10%, 20%, 30%, and 40%. The neural health and current spread settings correspond to Scenario 2, as described in Table 2.

The clean ECAP matrix is mixed with the measured noise at densities of 10%, 20%, 30%, and 40%. The average RMSE results are shown in Figure 15.

Figure 15a shows that the above preprocessing approaches can reduce RMSE by 15% compared to the unprocessed ECAP data (Unpro). The nonlinear-based denoising mask estimation employed by PECAP-CNN results in larger distortion than the other three preprocessing algorithms. The performance rankings of the experimental results are consistent with those shown in Figure 12. The rankings for ${\bar{\epsilon }}_{M}$ are as follows: PECAP-TSPD (2.000%), PECAP-I-Median (4.890%), PECAP (6.113%), PECAP-CNN (8.850%), and Unpro (24.030%). For the neural activity pattern analysis presented in Figure 15b, the RMSE results for ${\bar{\epsilon }}_{A}$ can be ranked as PECAP-TSPD (1.311%), PECAP-I-Median (3.657%), PECAP (5.405%), and PECAP-CNN (6.585%). The results of TDCC and SSIM are described in Table 9.

Table 9 shows a similar trend to that in Figure 15, where the average TDCC performance ranking is as follows: PECAP-TSPD, PECAP-Median, PECAP, and PECAP-CNN. The average SSIM rank performance is as follows: PECAP-TSPD, PECAP-I-Median, PECAP-CNN, and PECAP. From Figure 12 and Figure 15 (i.e., the simulated and experimental results), the average RMSEs of ECAP magnitudes and auditory neural activity patterns can be ranked as follows: PECAP-TSPD (1.952%, 1.407%), PECAP-I-Median (4.515%, 3.3215%), PECAP (6.446%, 5.7035%), and PECAP-CNN (9.700%, 7.111%). Similarly, the average TDCC and SSIM from Table 6 and Table 9 can be ranked as follows: PECAP-TSPD (0.9988, 0.9931), PECAP-I-Median (0.9949, 0.9470), PECAP (0.9859, 0.8997), and PECAP-CNN (0.9766, 0.8832). These results show that the proposed TSPD algorithm performs well under random noise conditions (Figure 13 and Table 7) and has robust impulse noise resistance.

## 5. Conclusions

The PECAP-TSPD algorithm, which integrates an improved spatial median filter, the log-spectral amplitude Wiener filter, and the PECAP framework, was developed to reduce noise in ECAP data and enable more accurate estimation of ECAP magnitudes and auditory neural activity patterns from severely corrupted ECAP matrices. A reordering technique was proposed based on the physiological characteristics of ECAP signals to assist LSA Wiener filtering in the second denoising stage, aiming to estimate the noise region of the ECAP matrix. The effectiveness of this estimation was verified using the proposed CNN-based denoising mask. Quantitative evaluations using normalized root mean square error (RMSE) for ECAP magnitude ($\epsilon_{M}$) and auditory neural activity pattern ($\epsilon_{A}$) revealed that both PECAP and PECAP-TSPD significantly reduce error metrics compared to unprocessed data across various signal-to-noise ratios (SNRs), noise densities, and test scenarios. PECAP-TSPD consistently outperformed PECAP in terms of both $\epsilon_{M}$ and $\epsilon_{A}$. For ECAP matrices contaminated by random noise, the average ${\bar{\epsilon }}_{M}$ values across seven scenarios and twelve SNR levels were as follows: PECAP-TSPD (3.83%), PECAP (5.14%), and unprocessed (23.07%). Under impulse noise, the corresponding values were as follows: PECAP-TSPD (3.13%), PECAP (5.57%), and unprocessed (21.97%). Similarly, the average ${\bar{\epsilon }}_{A}$ under random noise was 3.01% for PECAP-TSPD and 4.64% for PECAP, while, under impulse noise, the values were 2.39% and 5.23%, respectively. The simulated and experimental results also showed that the proposed TSPD algorithm performs best in terms of RMSE (${\bar{\epsilon }}_{M}=$ 1.952%, ${\bar{\epsilon }}_{A}=$ 1.407%), TDCC (0.9988), and SSIM (0.9931), when compared to the baselines (PECAP, PECAP-I-Median, and PECAP-CNN). Future work will include validating the proposed PECAP-TSPD algorithm using clinical ECAP data to assess its robustness and practical applicability in real-world auditory diagnostic contexts.

## Figures and Tables

**Table 1:** Steps of the LSA Wiener filtering algorithm [24,25,26].

Step 1. Initialization of $P_{u}\left(k\right)={\left|\frac{1}{N_{u}}\sum_{l=1}^{N_{u}}\left|Y_{m}(l,k)\right|\right|}^{2}$ for each frequency bin For each l and k: Step 2. Estimation of $\gamma \left(l,k\right)$ If l=1, then $\gamma \left(l,k\right)=\frac{{\left|Y_{m}(l,k)\right|}^{2}}{P_{u}\left(k\right)}$, else $\gamma \left(l,k\right)=\frac{{\left|Y_{m}(l,k)\right|}^{2}}{P_{u}\left(l-1,k\right)}$ Step 3. Estimation of $\hat{\xi }\left(l,k\right)$ using Equation (16) If l=1, then Equation (16) can be rewritten as $\hat{\xi }\left(l,k\right)=(1-\alpha )max\left\{\gamma \left(l,k\right)-1,0\right\}$ Step 4. check the VAD criterion If $\sum_{l=1}^{N_{t}}\Lambda \left(l,k\right)<\varepsilon_{1}$, then using Equation (20) for updating $P_{u}\left(l,k\right)$ Step 5. Calculation of $H_{LSA}\left(l,k\right)$ using Equation (15) Step 6. Calculation of $\hat{X}\left(l,k\right)$ using Equation (21) End for

**Table 2:** Neural health and current spread settings used in different scenarios.

Scenario 1: $\eta_{i}=1$, $\sigma_{i}=1.5$, i=1,2,…,N, where N=22 in this study. Scenario 2: $\eta_{i}=1$, $\sigma_{i}=2.5$, i=1,2,…,N. Scenario 3: $\eta_{i^{\prime }}=1$, $i^{\prime }=1,2,\ldots ,13,21,22$. $\eta_{14}=\eta_{20}=0.75$, $\eta_{15}=\eta_{19}=0.50$, $\eta_{16}=\eta_{18}=0.25$, $\eta_{17}=0.10$. $\sigma_{i}=1.5$, i=1,2,…,N. Scenario 4: $\eta_{i^{\prime }}=1$, $i^{\prime }=1,2,\ldots ,13,21,22$. $\eta_{14}=\eta_{20}=0.75$, $\eta_{15}=\eta_{19}=0.50$, $\eta_{16}=\eta_{18}=0.25$, $\eta_{17}=0.10$. $\sigma_{i}=2.5$, i=1,2,…,N. Scenario 5: $\eta_{i^{\prime }}=1$, $i^{\prime }=1,2,\ldots ,18$. $\eta_{19}=0.75$, $\eta_{20}=0.50$, $\eta_{21}=0.25$, $\eta_{22}=0.10$. $\sigma_{i}=1.5$, i=1,2,…,N. Scenario 6: $\eta_{i^{\prime }}=1$, $i^{\prime }=1,2,\ldots ,18$. $\eta_{19}=0.75$, $\eta_{20}=0.50$, $\eta_{21}=0.25$, $\eta_{22}=0.10$. $\sigma_{i}=2.5$, i=1,2,…,N. Scenario 7: $\eta_{i^{\prime }}=0.5$, $i^{\prime }=1,2,\ldots ,12,15,22$. $\eta_{13}=0.6$, $\eta_{14}=0.7$, $\eta_{16}=\eta_{21}=0.4$, $\eta_{17}=\eta_{20}=0.3$, $\eta_{18}=\eta_{19}=0.2.$ $\sigma_{1}=1.5$, $\sigma_{i^{\prime \prime }}=2.5-0.05\left(i^{\prime \prime }-1\right)$, $i^{\prime \prime }=2,\ldots ,N$

**Table 3:** RMSE results of PECAP and PECAP-TSPD under 16, 19, 22, and 25 SNRs. The neural health and current spread settings correspond to Scenario 1, as described in Table 2.

	SNR = 16 dB	SNR = 19 dB	SNR = 22 dB	SNR = 25 dB
PECAP	1.1842%	1.2858%	0.9113%	0.6459%
PECAP-TSPD	1.4958%	1.1209%	0.8892%	0.7239%

**Table 4:** Architecture of the proposed three-convolutional-layer network for noise reduction mask estimation.

Layer Name	Input Size	Hyperparameters	Output Size
Reshape	22×22		22×22×1
Conv 1	22×22×1	3×3×1×20	22×22×20
Conv 2	22×22×20	3×3×20×20	22×22×20
Conv 3	22×22×20	3×3×20×1	22×22×1
Reshape	22×22×1		22×22

**Table 5:** TDCC and SSIM results of the CNN-based denoising mask for the clean ECAP matrix and the noisy ECAP matrix.

TDCC		
	Clean ECAP matrix	Noisy ECAP matrix
CNN mask	**0.9553**	0.8888
SSIM		
	Clean ECAP matrix	Noisy ECAP matrix
CNN mask	**0.5764**	0.3691

**Table 6:** Average TDCC and SSIM results of PECAP-CNN, PECAP, PECAP-I-Median, and PECAP-TSPD under impulse noise conditions with densities of 10%, 20%, 30%, and 40%.

	PECAP-CNN	PECAP	PECAP-I-Median	PECAP-TSPD
Average TDCC	0.9733	0.9855	0.9959	**0.9988**
	PECAP-CNN	PECAP	PECAP-I-Median	PECAP-TSPD
Average SSIM	0.8727	0.9166	0.9678	**0.9929**

**Table 7:** Average TDCC and SSIM results of PECAP-CNN, PECAP, PECAP-I-Median, and PECAP-TSPD under random noise conditions with SNRs of −5 dB, −2 dB, 1 dB, 4 dB, 7 dB, 10 dB, 13 dB, 16 dB, 19 dB, 22 dB, 25 dB, and 100 dB.

	PECAP-CNN	PECAP	PECAP-I-Median	PECAP-TSPD
Average TDCC	0.9620	0.9709	0.9805	**0.9993**
	PECAP-CNN	PECAP	PECAP-I-Median	PECAP-TSPD
Average SSIM	0.8566	0.8744	0.8709	**0.9952**

**Table 8:** TDCC and SSIM results of PECAP-TSPD with rectangular windows of lengths 11, 22, and 44. SNR = −5 dB.

	$N_{w}=11$	$N_{w}=22$	$N_{w}=44$
TDCC	0.9952	**0.9989**	0.9331
SSIM	0.9520	**0.9920**	0.5521

**Table 9:** Average TDCC and SSIM results of PECAP-CNN, PECAP, PECAP-I-Median, and PECAP-TSPD under measured noise conditions with densities of 10%, 20%, 30%, and 40%.

	PECAP-CNN	PECAP	PECAP-I-Median	PECAP-TSPD
Average TDCC	0.9799	0.9864	0.9940	**0.9988**
	PECAP-CNN	PECAP	PECAP-I-Median	PECAP-TSPD
Average SSIM	0.8938	0.8828	0.9262	**0.9934**

## Data Availability

The data supporting this study’s findings are available from the corresponding author upon reasonable request.

## Abbreviations

The following abbreviations are used in this manuscript:

ECAP	Electrically evoked compound action potential
PECAP	Panoramic ECAP
CI	Cochlear implant
SNR	Signal-to-noise ratio
TSPD	Two-stage preprocessing denoising algorithm
LSA	Log-spectral amplitude
RMSE	Root mean square error
Unpro	Unprocessed data
I-Median	Improved median filtering
CNN	Convolutional neural network
TDCC	Two-dimensional correlation coefficient
SSIM	Structural similarity index
